# Association Between Electrophysiological Evaluation and Muscle Quality Changes in the Lower Limb of Subacute Stroke Patients: A Pilot Longitudinal Observational Study

**DOI:** 10.3390/diagnostics15222854

**Published:** 2025-11-11

**Authors:** Se Jin Kim, Jeong Hwan Lee, Young Sook Park, Hyun Jung Chang, Jin Gee Park, Eun Sol Cho, Jae Yeon Kim, Dong Jin Ha

**Affiliations:** 1 Department of Physical Medicine and Rehabilitation, Samsung Changwon Hospital, Sungkyunkwan University School of Medicine, Changwon 51353, Republic of Korea; kimsj_12@naver.com (S.J.K.); jijibaeheiwon@daum.net (Y.S.P.); reh.chj@gmail.com (H.J.C.); jingee00@naver.com (J.G.P.); rlawodus21@gmail.com (J.Y.K.); haom77@naver.com (D.J.H.); 2 Department of Physical Medicine and Rehabilitation, Jalbon Hospital, Changwon 51320, Republic of Korea; orange200000@naver.com

**Keywords:** subacute stroke, shear wave elastography, electrophysiology, sarcopenia, muscle quality, gastrocnemius, vastus lateralis

## Abstract

**Background:** This pilot longitudinal observational study investigated 4-week changes in lower limb muscle quantity and quality in patients with subacute stroke and explored risk factors associated with these changes. **Methods:** Twenty-six patients with hemiplegia following subacute stroke underwent assessment at baseline and 4-week follow-up. Muscle quantity was evaluated by ultrasound muscle thickness and bioelectrical impedance analysis, while muscle quality was assessed by shear-wave elastography in seven muscles (rectus femoris, vastus intermedius, vastus lateralis [VL], vastus medialis, tibialis anterior, gastrocnemius [GCM], and soleus). Electrophysiological assessments included motor-evoked potential (MEP), somatosensory-evoked potential (SEP), nerve conduction studies (NCSs), and central motor conduction time (CMCT). **Results:** Muscle thickness and bioimpedance did not significantly change between baseline and follow-up. In contrast, shear modulus increased in the paretic-side VL and GCM muscles (*p* < 0.001 and *p* = 0.049), with no differences in the non-paretic side. Greater deterioration in GCM quality was observed in patients with abnormal lower-limb MEP, and increased VL stiffness correlated with prolonged CMCT. Multivariable analyses were performed adjusting for age, sex, National Institutes of Health Stroke Scale, and comorbidity burden; however, due to the small electrophysiology sample (*n* = 11), these results should be interpreted as exploratory. **Conclusions:** In subacute stroke, early deterioration in muscle quality can occur despite stable quantity and appears linked to corticospinal integrity. Integrating electrophysiological evaluation with elastography may help identify patients who could benefit from early, targeted neuromuscular rehabilitation. These exploratory findings require validation in larger cohorts.

## 1. Introduction

Stroke is a leading cause of disability worldwide and the second leading cause of death [1]. The global economic burden of stroke is estimated at USD 721 billion (0.66% of global GDP), and the cost of post-stroke disability continues to rise annually [1]. Accordingly, efforts have been made to improve functional outcomes in stroke survivors through early screening and rehabilitation initiatives. Early mobility and postural control are critical components of stroke rehabilitation, reducing fall risk and facilitating early functional recovery through improved sensorimotor coordination [2].

Following a stroke, muscle abnormalities can develop due to denervation, disuse, remodeling, and spasticity [3]. Age, physical inactivity, and poor nutrition further contribute to muscle wasting [4]. Because muscle wasting is closely linked to reduced gait speed and impaired sit-to-stand performance [5], non-invasive approaches have been actively developed to quantify such changes.

In chronic stroke, consistent evidence shows reductions in muscle quantity and deterioration in quality. Motor unit numbers decline first on the paretic side and later on the non-paretic side within one week, persisting into the chronic stage [6,7]. Cross-sectional and longitudinal studies report lower thigh muscle volume and increased intramuscular fat on the paretic side [7,8].

In contrast, evidence in subacute stroke remains inconsistent. Kim et al. reported decreased thickness of the VL and GCM on the paretic side, with increased echo intensity on both sides [9]. Another longitudinal study found no change in thickness but increased shear-wave modulus in paretic-side VL and GCM [10]. Similarly, higher shear-wave velocity was observed in the paretic-side biceps brachii [11]. These findings suggest that while qualitative changes occur early, quantitative loss in the subacute phase is less evident.

Risk factors for post-stroke sarcopenia include age, dyslipidemia, diabetes, ischemic heart disease, and nutritional status [12]. In subacute stroke, older age, tube feeding, high National Institutes of Health Stroke Scale (NIHSS), and reduced non-paretic calf circumference have been associated with muscle loss [13]. However, most previous studies have focused primarily on muscle quantity, offering limited insight into muscle quality during the subacute stage.

Recent meta-analytic evidence has highlighted that sensorimotor and neuromechanical rehabilitation approaches improve post-stroke motor recovery and gait performance [14], emphasizing the need for integrated assessment of muscle quantity and neural integrity. Recent neural rehabilitation frameworks have also highlighted the role of sensory cueing in shaping neuromotor control and supporting the integration of electrophysiological and biomechanical assessments in stroke rehabilitation [15]. This approach may enhance clinical decision-making by providing a neurophysiological rationale for personalized rehabilitation planning.

This pilot longitudinal study therefore aimed to evaluate 4-week changes in lower limb muscle quantity and quality in patients with subacute stroke. Unlike our previous work focusing solely on morphological parameters [10], the present study integrates electrophysiological markers to link neural integrity with muscle-quality alterations. By combining electrophysiological and elastographic measures, this study provides a mechanistic understanding of how corticospinal integrity relates to peripheral muscle adaptation, offering a neurophysiological basis for individualized rehabilitation planning and early clinical decision-making. This integration provides new insight beyond our previous work, which focused solely on morphological parameters. Furthermore, recent systematic reviews on ultrasound elastography and sarcopenia [16,17] were incorporated to contextualize our findings within the broader literature.

## 2. Materials and Methods

### 2.1. Study Design and Participants

The reporting of this study followed the STROBE (Strengthening the Reporting of Observational Studies in Epidemiology) guidelines, as recommended by the EQUATOR Network. This pilot observational longitudinal study enrolled patients hospitalized with unilateral ischemic or hemorrhagic stroke between September 2022 and April 2023.

Of 36 patients screened, 26 completed all assessments and were included in the final analysis, while 10 were excluded owing to early discharge (*n* = 6), or medical complications (*n* = 4). During their rehabilitation at the Department of Rehabilitation Medicine, all participants received conventional therapy twice daily, five days per week. Conventional rehabilitation consisted of two 30-min sessions of physical and occupational therapy per day, focusing on neurodevelopmental facilitation, task-oriented training, strengthening of paretic limbs, and gait re-education.

Among the included patients, 16 (61.5%) had supratentorial and 10 (38.5%) had infratentorial lesions. Twenty (76.9%) had ischemic and six (23.1%) hemorrhagic stroke. Because of the exploratory nature and limited number of eligible inpatients during the study period, the sample size was determined by feasibility rather than a priori power calculation, and the cohort was thus considered a pilot study.

Inclusion criteria were (1) adults aged ≥19 years with first-ever unilateral stroke confirmed by computed tomography or magnetic resonance imaging; (2) ambulatory prior to stroke onset; and (3) provision of written informed consent. Exclusion criteria were (1) pre-existing limitations in walking or balance; (2) neuromuscular, renal, orthopedic, or uncontrolled psychiatric disorders; (3) inability to undergo body-composition or electrophysiological testing due to pacemaker or metallic implants; (4) clinically significant focal entrapment neuropathies (e.g., carpal tunnel or peroneal nerve palsy); (5) cognitive impairment interfering with compliance; (6) severe visual or hearing impairment; and (7) refusal to participate.

All procedures were conducted in accordance with the Declaration of Helsinki and approved by the Institutional Review Board of by Samsung Changwon Hospital, Sungkyunkwan University School of Medicine (protocol code SCMC 2022-07-003; approval date: 10 August 2022). Written informed consent was obtained from all participants.

This pilot observational study was not registered in a public trial registry, because it did not involve interventional procedures.

### 2.2. Data Collection

Clinical characteristics, including age, sex, education level, comorbidities, height, weight, and dietary habits, were recorded. Laboratory parameters included C-reactive protein (mg/dL) and glycated hemoglobin (HbA1c, %). Stroke severity was assessed using the NIHSS, and cognitive function was evaluated with the Mini-Mental State Examination (MMSE). Comorbidities were quantified using the Charlson Comorbidity Index [18].

Nutritional status was evaluated using the Geriatric Nutritional Risk Index (GNRI), an index for assessing malnutrition, calculated as follows:

GNRI = (1.487 × serum albumin level [g/L]) + (41.7 × body weight/ideal body weight),

where ideal body weight (kg) = height^2^ (m^2^) × 22 [19].

Heavy drinking was defined as consumption of >15 drinks per week for men and >8 drinks per week for women, in accordance with the Centers for Disease Control and prevention (CDC) criteria [20]. Calf circumference on the non-paretic side was measured to the nearest 1 cm at the thickest part of the calf, with the knee flexed at 90° in the supine position. The measurement site was standardized at 30% of the distance between the lateral malleolus and the lateral tibial condyle [21].

### 2.3. Bioelectrical Impedance Analysis

Bioelectrical impedance analysis (BIA) was performed using an InBody S10 device (InBody Co., Ltd., Seoul, Republic of Korea). Measurements were obtained at baseline and at the 4-week follow-up during the same session as the ultrasound and electrophysiological assessments, following a standardized electrode protocol recommended by the manufacturer.

Parameters included total fat mass, fat mass index, appendicular skeletal muscle mass (ASM), body fat percentage, and their derived indices: fat mass index (FMI = fat mass [kg]/height^2^ [m^2^]) and fat-free mass index (FFMI = fat-free mass [kg]/height^2^ [m^2^]).

All BIA measurements were taken after at least 8 h of fasting, with participants in the supine position and both limbs slightly abducted to minimize impedance error.

### 2.4. Ultrasonographic Measurements

Ultrasound examinations were performed using a multi-frequency linear transducer (8–12 MHz) on a B-mode ultrasound system (Samsung Medison V8; Samsung Medison, Seoul, Republic of Korea). Muscle thickness and elasticity of the rectus femoris (RF), vastus intermedius (VI), VL, vastus medialis (VM), tibialis anterior (TA), GCM, and soleus were measured bilaterally.

RF and VI were measured midway between the anterior superior iliac spine and the proximal patella; VM at 12.5% medial to 20% of this distance; VL at 10% lateral to the midpoint; TA at 30% proximal between the lateral malleolus and lateral tibial condyle; and GCM/soleus at 30% proximal between the lateral malleolus and lateral tibial condyle [7,10]. Measurements for the GCM and soleus were obtained in the sitting position, and all other muscles were examined in the supine position.

All measurements were performed with the probe placed perpendicular to the skin surface, applying minimal compression and using an ample amount of gel to minimize anisotropy artifacts.

**Figure 1:**
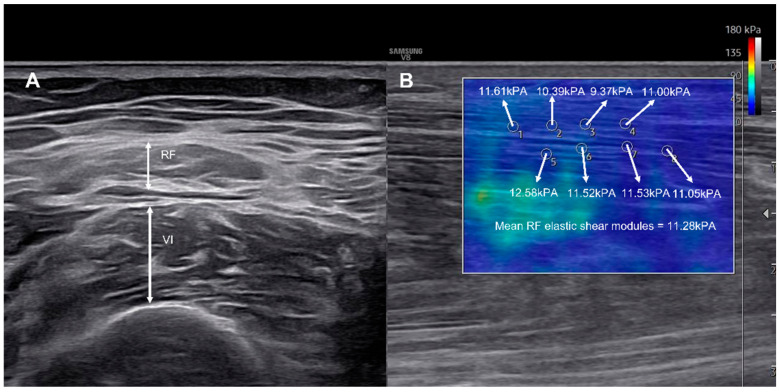
Representative ultrasonographic images showing measurement of RF and VI muscle thickness (**A**), and corresponding shear-wave elastography map (**B**). The color scale indicates the elastic shear modulus (kPa) ranging from 0 to 180 where blue represents softer tissue and red indicates stiffer tissue. Multiple regions of interest (ROIs) were positioned along the muscle fascicles, and mean shear modulus was automatically calculated by the system.

Muscle thickness was recorded to the nearest 0.01 cm using electronic calipers. Shear-wave elastography was used to assess viscoelastic properties, with the probe aligned longitudinally along the muscle fascicles [22]. A circular region of interest (ROI, 1 mm diameter) was placed at the mid-belly of each muscle, and the mean value of eight ROIs was calculated as the elastic shear modulus (kPa) [10,23] (Figure 1). Each measurement was performed twice by the same experienced operator, and the average value was used for analysis to ensure intra-rater reliability.

### 2.5. Electrophysiological Examination

Electrophysiological testing included MEP, SEP, and NCSs. A Nicolet AT2+6 amplifier (Natus Medical Inc., Middleton, WI, USA) and a MagPro simulator (MagVenture A/S, Farum, Denmark) were used. All studies were performed by a single experienced physician. All electrophysiological and ultrasonographic assessments were conducted by a board-certified physiatrist with over 10 years of clinical experience, and all rehabilitation sessions were administered by licensed physical therapists with more than 5 years of experience in neurorehabilitation. Magnetic cortical stimulation was not performed in patients with metallic devices or those at risk of seizure.

Bilateral MEP recordings were obtained from the abductor pollicis brevis (APB) in the upper limb and the abductor hallucis (AH) in the lower limb using surface electrodes with participants in the supine position. Transcortical stimulation was delivered at 60% of the maximum simulator output using a figure-8 coil. The stimulus duration was 0.5 ms, with a filter of 3–10 Hz and sweep speed of 5 ms for both limbs. MEP latency and amplitude were analyzed. Responses were classified as abnormal if MEPs were absent, latency was prolonged (≥21.7 ms for the upper limb, ≥41.5 ms for the lower limb), or amplitude was reduced more than 50% compared with the contralateral side (*n* = 11 and *n* = 9, respectively) [24,25].

SEP responses were elicited by electrical stimulation of the median nerve at the wrist and the posterior tibial nerve at the ankle (7.5 mA for the upper limb, 8.0 mA for the lower limb). The stimulus duration was 0.2 ms and frequency 3.1 Hz. Cortical responses were recorded using surface electrodes placed over contralateral C3’/C4’, referenced to Cpz’, according to the 10–20 system. Recordings were filtered 30–3000 Hz. N17 and P21 latencies were measured for the upper limb, P42 and N50 for the lower limb. SEP abnormalities were defined as latency prolongation beyond normal limits. (≥20.63 ms, ≥28.23 ms, ≥42.6 ms, and ≥51.8 ms, respectively [24].

Motor NCSs were performed on the bilateral median, ulnar, peroneal, and posterior tibial nerves to assess latency, amplitude, and conduction velocity. Sensory NCSs were conducted on the bilateral median, ulnar, superficial peroneal, and sural nerves to measure latency, amplitude, and sensory nerve action potential. Peripheral polyneuropathy was diagnosed and graded using Baba classification(0–4) [26].

Peripheral motor conduction time (PMCT) was calculated from F-wave and M-wave latencies recorded at the APB and AH muscles using the equation (F + M − 1)/2 [27]. CMCT was calculated as:

CMCT = MEP − (F + M − 1)/2

for the upper limb (*n* = 10) and lower limb (*n* = 2) recordings [24]. Because the lesion site (supratentorial vs. infratentorial) can influence conduction latency, results were interpreted in the context of each patient’s lesion location.

### 2.6. Statistical Analysis

Longitudinal changes in muscle thickness, elastic shear modulus, and body composition were analyzed using the Wilcoxon signed-rank test. The normality of continuous variables was examined using the Shapiro–Wilk test, confirming non-normal distribution.

Electrophysiological testing was performed in 21 participants; however, due to absent or unobtainable MEP or F-wave responses, CMCT could not be calculated in 10 patients, leaving 11 participants with complete electrophysiological datasets for regression analysis. Multivariable linear regression adjusting for age, sex, NIHSS, and Charlson Comorbidity Index (CCI) was performed in the full cohort (*n* = 26). For models including electrophysiological variables (e.g., CMCT, MEP, SEP), the number of complete cases was small (*n* = 11), yielding fewer than 10 events per variable. Therefore, multivariable adjustment was considered statistically underpowered and was not performed. Univariable analyses were reported as exploratory, and beta coefficients, standard errors, and 95% confidence intervals (CIs) were presented. Given the small electrophysiology subgroup (*n* = 11), these analyses were statistically underpowered and should be interpreted as hypothesis-generating.

All analyses were conducted using SPSS Statistics, version 21.0 (IBM Corp., Armonk, NY, USA). Effect sizes (*r* = *Z*/√*N*) were calculated for Wilcoxon tests to indicate the magnitude of change. Given the small sample size, Type II errors could not be excluded. Statistical significance was set at *p* < 0.05. A post hoc power analysis (G*Power v3.1; two-tailed, α = 0.05) for the primary outcome (change in paretic VL shear modulus) was conducted using a paired-sample design. The analysis yielded a Cohen’s dz of 0.76 and a statistical power (1 − β) of 0.959, indicating adequate sensitivity despite the small sample size.

## 3. Results

### 3.1. Demographic Characteristics

**Figure 2:**
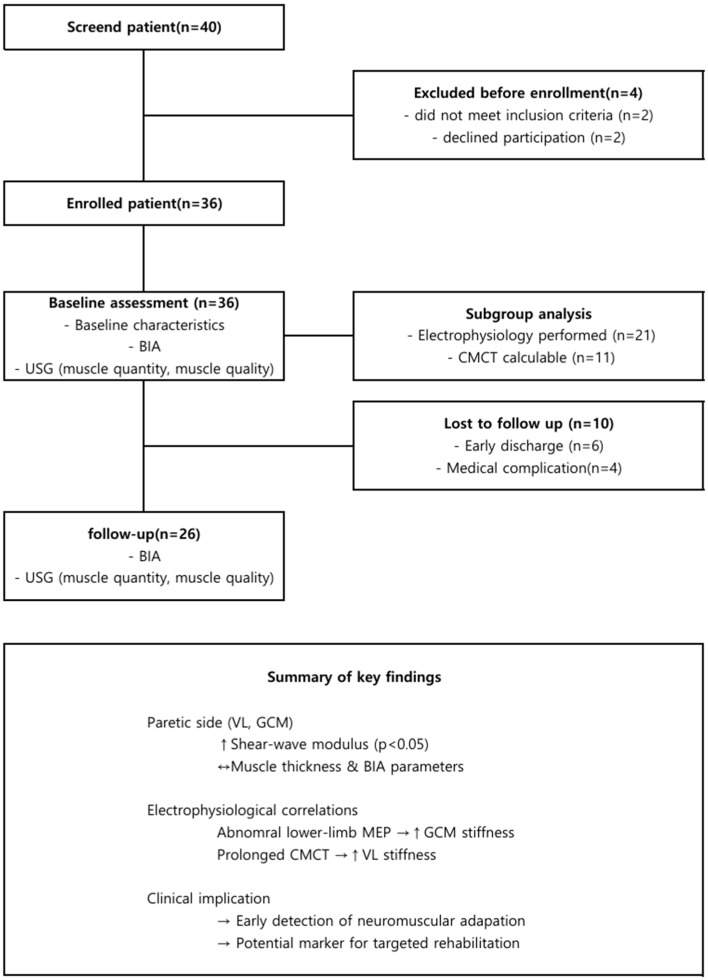
Participant flow and summary of main findings. (**Top**): study flow; (**Bottom**): key quantitative outcomes summarized in a table. ↑, increase; →, causal relationship; ↔, no difference.

A total of 36 patients were screened, and 26 completed all assessments and were included in the final analysis, while 10 were excluded due to early discharge (*n* = 6), or medical complications (*n* = 4) (Figure 2).

The final cohort comprised 9 men and 17 women, with a median age of 72 years (IQR 61–80). The median time since stroke onset was 7 days (range, 4–11). Stroke type distribution included 20 ischemic and 6 hemorrhagic cases. Lesion location consisted of 16 supratentorial and 10 infratentorial cases. No significant between-group differences were observed in 4-week changes in shear modulus according to stroke type (ischemic vs. hemorrhagic), age group or lesion location (supratentorial vs. infratentorial). (all *p* > 0.05). Education level was recorded as an indicator of cognitive reserve that may influence rehabilitation compliance. Demographic variables are summarized in Table 1.

### 3.2. Bioelectrical Impedance Analysis

BIA showed no significant differences between baseline and 4-week follow-up in any body composition parameter, including fat mass, fat mass index, fat-free mass, fat-free mass index, skeletal muscle mass, body fat percentage, appendicular skeletal muscle mass/height^2^ (ASM/height^2^), or body weight (all *p* > 0.05) (Table 2).

### 3.3. Ultrasonographic Assessment

Muscle thickness across the seven examined muscles showed no significant differences between baseline and 4-week follow-up on either the paretic or non-paretic side (all *p* > 0.05; Table 3). In contrast, shear-wave elastography revealed significant increases in the elastic shear modulus of the paretic VL and GCM muscles (*p* < 0.001 and *p* = 0.049, respectively), with no significant changes observed in non-paretic muscles (Table 4).

### 3.4. Regression Analysis

Univariable regression analysis showed no significant associations between demographic variables and changes in shear-wave modulus of the paretic VL and GCM muscles (Table 5). In multivariable models adjusting for age, sex, NIHSS, and CCI, the direction and statistical significance of the associations remained unchanged (Appendix A Table A1). No additional significant predictors, including lesion location (supratentorial vs. infratentorial) or stroke type (ischemic vs. hemorrhagic), were identified.

### 3.5. Electrophysiologic Correlations

Among the 21 patients who underwent electrophysiological testing, significant differences were observed in shear-wave modulus changes in the paretic GCM muscle between the abnormal and normal lower-limb MEP groups (*p* = 0.039). Subgroup analysis of 10 upper-limb and 12 lower-limb patients (excluding cases with unobtainable MEP or F-wave measurements) revealed a significant association between increased lower-limb CMCT and shear-wave modulus changes in the paretic VL muscle (*p* = 0.032).

Because electrophysiology-complete cases were limited (*n* = 11), these associations between shear-wave modulus changes and CMCT/MEP/SEP were analyzed an univariable estimates only. Multivariable modeling was not performed due to instability of estimates and the risk of model overfitting with such a small sample size.

Clinically, greater muscle stiffness in paretic-side VL and GCM muscles was associated with impaired corticospinal conduction, suggestion that shear-wave elastography may serve as a complementary marker of post-stroke neuromuscular adaption. No significant correlations were found with upper-limb MEP abnormalities, SEP abnormalities, Baba classification, PMCT, or changes in GCM muscle quality (Table 6).

## 4. Discussion

This longitudinal study of patients with subacute stroke demonstrated that, while lower-limb muscle quantity remained stable over four weeks, muscle quality significantly deteriorated in the paretic VL and GCM. Importantly, these changes were not associated with established risk factors for stroke-related sarcopenia but were instead linked to electrophysiological findings, particularly abnormal lower-limb MEPs and prolonged CMCT. These results highlight the dynamic interplay between peripheral muscle mechanical properties and corticospinal excitability, suggesting that shear-wave elastography may serve as a complementary marker of neuromuscular adaptation after stroke [11,28,29].

Decreases in skeletal muscle mass are well known to increase the risk of falls, disability, reduced quality of life, and mortality [16]. As the social and clinical burden of muscle wasting continues to rise, early detection is crucial [30]. Beyond muscle mass, recent studies have emphasized the prognostic value of muscle quality, with poorer quality being associated with higher 90-day mortality after stroke [17]. Furthermore, recent meta-analyses have underscored that neuromechanical and sensorimotor retraining interventions can improve post-stroke gait and strength recovery, reinforcing the importance of integrating neural and muscular assessment in rehabilitation [15,17].

Conventional approaches such as DXA or BIA primarily assess muscle mass, whereas CT, although considered the gold standard, is limited by its high cost and radiation exposure [17]. Accordingly, ultrasonography and shear-wave elastography are increasingly used as non-invasive tools to evaluate both muscle quantity and quality.

Previous studies on chronic stroke have consistently demonstrated longitudinal reductions in muscle mass and quality [8,9]. In contrast, findings in subacute stroke have been less consistent, although deterioration in muscle quality tends to be more pronounced than that in quality [10,11,12]. In our cohort, BIA and ultrasound-derived muscle thickness showed no significant changes, whereas the elastic modulus increased in the paretic VL and GCM, confirming early deterioration in muscle quality.

Pathophysiologically, hemiplegic stroke leads to rapid skeletal muscle changes driven by disuse, denervation, and remodeling. Structural adaptations may begin within hours, and limb muscle loss emerges within the first week, accompanied by reductions in motor unit numbers [31]. Prior studies have demonstrated increased echo intensity (EI) in multiple muscles of chronic stroke patients compared with healthy controls [32], and similar increases in paretic VL and GCM in subacute patients [10]. Muscle-specific differences in adaptation are thought to arise from variations in anatomical attachment and immobilization-induced loading, and non-uniform patterns of atrophy have been reported, sparing certain muscles such as the TA and gracilis [33].

Risk factors for post-stroke sarcopenia—such as advanced age, comorbidities, high NIHSS, and reduced non-paretic calf circumference—have been well described [13,14]. However, these studies have largely addressed muscle quantity rather than quality. Our results suggest that such conventional risk factors may not adequately predict early deterioration in muscle quality, which appears to be more directly associated with electrophysiological impairment.

Electrodiagnostic testing is widely used in stroke to assess motor and sensory integrity and detect comorbid neuropathies. In this study, muscle quality deterioration was more pronounced in patients with abnormal lower-limb MEPs, and prolonged CMCT correlated with worsening VL quality. These findings are consistent with previous reports showing reduced MEP amplitudes in association with muscle weakness [34,35]. This link between central motor conduction delay and peripheral stiffness suggest that electrophysiological evaluation could help predict the degree of secondary myogenic adaptation and guide early, targeted rehabilitation strategies [11,28,29]. Recent neurophysiological analyses have shown that post-stroke motor recovery is associated with plastic changes in cortical excitability and sensorimotor network reorganization [29,35], complementing the current findings linking peripheral stiffness with central conduction parameters. These findings align with emerging evidence linking neurophysiological modulation and sensory–motor integration in stroke recovery, including peripheral sensory mechanisms and proprioceptive enhancement via taping [6,36]. Recent advances in SEP pattern analysis, such as the classification approach proposed by Fuseya et al. [6], may further enhance cortical sensory assessment in subacute stroke.

From a clinical perspective, early recognition of muscle quality decline is crucial, as it directly affects functional prognosis, hospital stay, and quality of life. Integrating electrophysiological and shear-wave elastography assessments may enhance individualized rehabilitation planning and monitoring in patients with subacute stroke. Comprehensive rehabilitation outcome frameworks emphasizing sensorimotor performance may facilitate translation of muscle-quality metrics into individualized clinical decision-making [37]. These perspectives reinforce the clinical importance of proprioceptive and neuromechanical interventions for restoring lower-limb function after stroke [38]. This concept is supported by recent frameworks emphasizing integration of neural and muscular assessments for individualized rehabilitation [15,39].

This study has several limitations. First, the overall sample size was small (*n* = 26), and only 11 participants had complete electrophysiological data, multivariable adjustment including these variables would violate sample-per-variable requirements. Including these variables in regression models would have violated the minimum sample-per-variable requirement and produced unstable estimates. Therefore, the electrophysiological results should be regarded as exploratory and require validation in larger cohorts. Second, all participants underwent standardized rehabilitation, which precluded assessment of natural disease progression. Third, the cohort consisted predominantly of older adults, so the findings may not generalize to younger populations. Additionally, the 4-week interval offers only short-term information, and no healthy control group was included, these pilot findings provide a rationale for larger longitudinal studies incorporating electrophysiological and elastographic markers to track neuromuscular adaptation. Although the cohort was predominantly female and ischemic, sex and etiology-adjusted regression did not reveal significant interaction effects. Future studies could incorporate proprioceptive and motor-retraining approaches shown to enhance neuromuscular activation and stability in lower-limb rehabilitation. In addition, post-stroke fatigue, which can be categorized into distinct subtypes with varying contributing factors, may influence neuromuscular recovery. Recent findings by Etoom et al. [39] highlight the importance of recognizing these subtypes when tailoring rehabilitation strategies. Future multicenter studies with larger and more diverse populations are warranted to validate these results and to explore targeted intervention strategies.

## 5. Conclusions

In patients with subacute stroke, muscle quality of the paretic VL and GCM deteriorated significantly compared with the non-paretic side, despite stable muscle quantity. These alterations were not associated with conventional risk factors for stroke-related sarcopenia but were instead linked to electrophysiological abnormalities. Specifically, abnormal lower-limb MEPs were associated with greater deterioration in GCM quality, and prolonged CMCT correlated with worsening VL quality. These findings suggest that electrophysiological assessment, when combined with shear-wave elastography, may serve as a complementary tool for detecting and predicting early neuromuscular alterations in subacute stroke.

## Figures and Tables

**Table 1:** Baseline characteristics of the study population.

Variable	Patient(*n* = 26)
Age, year, median (IQR)	72.00 (61.00–80.00)
Sex, *n* (%)	
Male	9 (34.62)
Female	17 (65.38)
Reason for admission, *n* (%)	
Infarction	20 (76.92)
Hemorrhage	6 (23.08)
Lesion location	
Supratentorial	16 (61.5)
Infratentorial	10 (38.5)
NIHSS, median (IQR)	7.00 (4.00–11.00)
Stroke duration, day, median (IQR)	9.00 (6.00–12.00)
MMSE, median (IQR)	20.00 (13.00–25.00)
Education, *n* (%)	
Un-educated	5 (19.23)
Elementary school	6 (23.08)
Middle school	3 (11.54)
High school	8 (30.77)
College	4 (15.38)
CCI, median (IQR)	30.25 (29.00–34.00)
GNRI, median (IQR)	102.73 (96.89–107.87)
Calf circumference, median (IQR) (cm)	30.25 (29.00–34.00)
Diet, *n* (%)	
Oral feeding	20 (76.92)
L-tube feeding	6 (23.08)
Diabetes mellitus, *n* (%)	9 (34.62)
Heavy drinking, *n* (%)	8 (30.77)
HbA1c, median (IQR) (%)	5.95 (5.50–6.70)
CRP, median (IQR) (mg/dL)	0.14 (0.07–0.31)

Data are presented as median (interquartile range) or frequencies (percentages), NIHSS = National Institutes of Health Stroke Scale; MMSE = Mini-Mental State Examination; CCI = Charlson Comorbidity Index; GNRI = Geriatric Nutritional Risk Index; CRP = C-reactive protein.

**Table 2:** Bioelectrical impedance analysis at baseline and follow-up (*n* = 26).

Variable	Baseline	Follow-Up	Δ	*p*-Value
Fat mass (kg)	12.10 (5.90–16.00)	11.30 (7.60–16.90)	−0.80	0.872
Fat mass index (kg/m^2^)	4.89 (1.97–6.83)	4.58 (2.54–7.05)	−0.31	0.851
Fat-free mass(kg)	45.60 (36.60–55.90)	46.50 (40.80–54.30)	+0.90	0.798
Fat-free mass index (kg/m^2^)	18.15 (17.10–19.78)	18.36 (16.20–18.80)	+0.21	0.936
Skeletal muscle mass (kg)	24.85 (19.40–31.30)	24.80 (21.60–31.30)	−0.05	0.979
Percentage body fat (%)	19.00 (9.40–27.00)	20.50 (12.80–29.50)	+1.50	0.628
ASM/height^2^	8.19 (6.90–9.25)	7.88 (6.76–8.60)	−0.31	0.590
Weight (kg)	57.50 (52.00–63.00)	56.00 (50.50–63.00)	−1.50	0.989

Values are presented as median (interquartile range). *p*-values were obtained using the Wilcoxon signed-rank test. Δ represents median change (Follow-up − Baseline). *p*-values were obtained using the Wilcoxon signed-rank test. ASM = appendicular skeletal muscle mass.

**Table 3:** Changes in muscle thickness at baseline and follow-up (*n* = 26).

	Paretic Side (cm)				Non-Paretic Side (cm)			
	Baseline	Follow-Up	Δ	*p*-Value	Baseline	Follow-Up	Δ	*p*-Value
RF	0.53 (0.42–0.70)	0.54 (0.42–0.76)	+0.01	0.593	0.59 (0.55–0.75)	0.59 (0.48–0.78)	+0.00	0.703
VI	0.61 (0.47–0.80)	0.62 (0.47–0.94)	+0.01	0.619	0.75 (0.63–0.93)	0.69 (0.52–0.96)	−0.06	0.416
VL	1.25 (0.97–1.57)	1.25 (0.94–1.58)	+0.00	0.638	1.40 (1.19–1.57)	1.34 (1.15–1.51)	−0.07	0.527
VM	1.48 (1.25–1.78)	1.57 (1.25–1.78)	+0.09	0.581	1.50 (1.20–1.67)	1.53 (1.42–1.82)	+0.03	0.346
TA	2.33 (2.05–2.61)	2.32 (2.09–2.73)	−0.01	0.077	2.29 (2.11–2.53)	2.44 (2.13–2.63)	+0.15	0.077
GCM	1.17 (1.08–1.36)	1.10 (0.95–1.44)	−0.07	0.638	1.12 (1.03–1.35)	1.13 (1.00–1.43)	+0.01	0.869
Soleus	1.39 (0.95–1.71)	1.32 (1.13–1.43)	−0.07	0.500	1.58 (1.18–1.71)	1.54 (1.28–1.82)	−0.04	0.660

Values are presented as median (interquartile range). *p*-values were obtained using the Wilcoxon signed-rank test. Δ represents median change (Follow-up − Baseline). *p*-values were obtained using the Wilcoxon signed-rank test. RF = rectus femoris; VI = vastus intermedius; VL = vastus lateralis; VM = vastus medialis; TA = tibialis anterior; GCM = gastrocnemius.

**Table 4:** Changes in elastic shear modules at baseline and follow-up (*n* = 26).

	Paretic Side (kPa)				Non-Paretic Side (kPa)			
	Baseline	Follow-Up	Δ	*p*-Value	Baseline	Follow-Up	Δ	*p*-Value
RF	37.37 (28.21–61.59)	38.00 (29.62–54.84)	+0.63	0.869	37.40 (29.06–50.91)	50.29 (38.75–63.60)	+12.89	0.059
VI	44.63 (29.86–67.57)	45.35 (27.27–61.53)	+0.72	0.269	53.06 (39.96–71.79)	57.87 (44.11–74.43)	+4.51	0.424
VL	20.82 (13.20–28.21)	29.54 (18.31–43.25)	+8.72	<0.001 ^†^	19.91 (13.00–41.02)	17.54 (14.07–36.81)	−2.37	0.849
VM	16.95 (10.84–22.66)	17.99 (10.77–32.16)	+1.04	0.869	18.69 (9.12–31.29)	14.95 (11.48–23.73)	−3.74	0.316
TA	34.71 (26.02–54.22)	46.46 (30.78–55.71)	+11.75	0.131	41.54 (32.81–51.19)	41.89 (32.56–51.61)	+0.35	0.970
GCM	19.22 (16.65–23.13)	22.50 (17.05–37.94)	+3.30	0.049 ^†^	23.53 (17.45–33.22)	20.87 (14.04–26.87)	−2.66	0.367
Soleus	32.47 (20.82–43.19)	27.84 (20.67–46.11)	−4.63	0.585	31.71 (23.48–38.41)	38.38 (26.66–51.14)	+6.67	0.131

Values are presented as median (interquartile range). *p*-values were obtained using the Wilcoxon signed-rank test. † *p* < 0.05 indicates statistical significance. Δ represents median change (Follow-up − Baseline). *p*-values were obtained using the Wilcoxon signed-rank test. RF = rectus femoris; VI = vastus intermedius; VL = vastus lateralis; VM = vastus medialis; TA = tibialis anterior; GCM = gastrocnemius.

**Table 5:** Univariable linear regression of changes in shear-wave modulus of the paretic VL and GCM muscles and baseline characteristics.

Variable	Delta VL (kPa)		Delta GCM (kPa)	
		*p*-Value		*p*-Value
Age	−0.05 ± 0.18	0.774	−0.01 ± 0.29	0.970
Sex				
Male	Reference		Reference	
Female	4.02 ± 5.36	0.461	−9.00 ± 8.46	0.298
Reason for admission				
Infarction	Reference		Reference	
Hemorrhage	6.46 ± 6.28	0.314	5.12 ± 10.18	0.619
Lesion location				
Supratentorial	Reference		Reference	
Infratentorial	4.45 ± 5.48	0.425	5.08 ± 8.80	0.569
NIHSS	0.72 ± 0.49	0.160	−0.48 ± 0.81	0.557
Stroke duration	−0.22 ± 0.37	0.563	−0.10 ± 0.60	0.872
MMSE	−0.09 ± 0.31	0.777	0.53 ± 0.49	0.285
Education				
Un-educated	Reference		Reference	
Elementary school	−1.89 ± 8.82	0.832	−19.02 ± 13.20	0.164
Middle school	4.92 ± 10.63	0.648	−12.53 ± 15.92	0.440
High school	1.77 ± 8.30	0.833	−21.65 ± 12.43	0.096
College	−0.86 ± 9.77	0.931	−11.57 ± 14.63	0.435
CCI	0.51 ± 1.28	0.693	−3.23 ± 1.94	0.109
GNRI	−0.22 ± 0.29	0.456	0.02 ± 0.47	0.968
Calf circumference	0.12 ± 0.78	0.874	−0.06 ± 1.24	0.962
Diet				
Oral feeding	Reference		Reference	
L-tube feeding	3.21 ± 6.39	0.620	0.30 ± 10.24	0.977
Diabetes mellitus	5.30 ± 5.58	0.352	−11.39 ± 8.76	0.206
Heavy drinking	2.40 ± 5.84	0.685	5.92 ± 9.27	0.529
HbA1c	−0.35 ± 1.95	0.859	−1.75 ± 3.09	0.577
CRP	−3.21 ± 3.30	0.338	2.11 ± 5.35	0.698

Values are standardized β coefficients (95% confidence interval) from univariable linear regression analysis. Abbreviations: NIHSS = National Institutes of Health Stroke Scale; MMSE = Mini-Mental State Examination; CCI = Charlson Comorbidity Index; GNRI = Geriatric Nutritional Risk Index; CRP = C-reactive protein.

**Table 6:** Univariable linear regression of changes in shear-wave modulus of the paretic VL and GCM muscles with electrodiagnostic parameters.

Variable	Delta VL (kPa)		Delta GCM (kPa)	
	β ± Se	*p*-Value	β ± Se	*p*-Value
SEP UEx (*n* = 21)	2.00 ± 5.92	0.739	12.87 ± 10.57	0.238
SEP LEx (*n* = 21)	4.06 ± 6.13	0.516	11.08 ± 11.16	0.333
MEP UEx (*n* = 21)	−2.87 ± 13.13	0.830	15.39 ± 24.02	0.529
MEP LEx (*n* = 21)	12.42 ± 6.54	0.073	25.99 ± 11.74	0.039 ^†^
CMCT UEx (*n* = 21)	2.09 ± 1.89	0.303	0.02 ± 3.73	0.996
CMCT LEx (*n* = 21)	2.87 ± 1.15	0.032 ^†^	−0.55 ± 2.73	0.844
PMCT UEx (*n* = 21)	1.11 ± 1.69	0.521	1.07 ± 3.15	0.739
PMCT LEx (*n* = 21)	1.02 ± 0.93	0.290	0.81 ± 1.76	0.651
Baba’s classification (*n* = 26)	−3.45 ± 3.32	0.310	−9.98 ± 5.02	0.058

Values are standardized β coefficients (95% confidence interval) from univariable linear regression analysis. † *p* < 0.05 indicates statistical significance. Abbreviations: SEP = somatosensory-evoked potential; UEx = upper extremity; LEx = lower extremity; MEP = motor-evoked potential; CMCT = central motor conduction time; PMCT = peripheral motor conduction time.

## Data Availability

The data presented in this study are available on request from the corresponding author. The data are not publicly available due to privacy and ethical restrictions.

## Abbreviations

MT	Muscle thickness
BIA	Bioelectrical impedance analysis
VL	Vastus lateralis
GCM	Gastrocnemius
MEP	Motor-evoked potential
CMCT	Central motor conduction time
NIHSS	National institutes of health stroke scale
MMSE	Mini-mental state examination
GNRI	Geriatric nutritional risk index
RF	Rectus femoris
VI	Vastus intermedius
VM	Vastus medialis
TA	Tibialis anterior
ROI	Region in interest
SEP	Sensory-evoked potential
PPN	Peripheral neuropathy

## Appendix A

**Table A1:** Multivariable linear regression of changes in shear-wave modulus of the paretic VL and GCM muscles with baseline characteristics.

Variable	Delta VL (kPa)			Delta GCM (kPa)		
	β ± Se	95% CI	*p*-Value	β ± Se	95% CI	*p*-Value
Age	0.53 ± 0.57	−0.69 to 1.76	0.364	−0.26 ± 0.37	−1.06 to 0.54	0.491
Sex						
Male	Reference			Reference		
Female	−16.86 ± 18.19	−55.88 to 22.16	0.370	11.33 ± 11.89	−14.16 to 36.82	0.356
Reason for admission						
Infarction	Reference			Reference		
Hemorrhage	11.56 ± 14.03	−18.54 to 41.66	0.424	6.64 ± 9.17	−13.03 to 26.30	0.481
Lesion location						
Supratentorial	Reference			Reference		
Infratentorial	−5.69 ± 12.57	−2.83 to 21.28	0.658	4.85 ± 8.21	−12.77 to 22.47	0.564
NIHSS	−0.24 ± 1.21	−2.83 to 2.35	0.847	0.75 ± 0.79	−0.95 to 2.44	0.361
Stroke duration	−0.54 ± 0.93	−2.53 to 1.45	0.571	−0.30 ± 0.61	−1.60 to 1.00	0.631
MMSE	0.71 ± 0.87	−1.16 to 2.59	0.429	0.13 ± 0.57	−1.10 to 1.35	0.830
Education						
Un-educated	Reference			Reference		
Elementary school	−13.93 ± 17.35	−51.14 to 23.59	0.436	0.10 ± 11.37	−24.21 to 24.42	0.993
Middle school	−0.33 ± 23.36	−50.86 to 50.20	0.989	3.83 ± 15.39	−29.19 to 36.84	0.807
High school	−25.57 ± 14.84	−57.40 to 6.25	0.107	2.43 ± 9.70	−18.36 to 23.23	0.806
College	−13.61 ± 21.22	−59.13 to 31.89	0.531	7.33 ± 13.86	−22.40 to 37.07	0.605
CCI	−5.53 ± 3.76	−13.46 to 2.40	0.159	1.16 ± 2.32	−3.29 to 6.52	0.497
GNRI	−0.07 ± 0.07	−0.22 to 0.07	0.310	0.06 ± 0.04	−0.03 to 0.15	0.171
Calf circumference	0.83 ± 1.04	−1.36 to 3.03	0.435	0.797 ± 0.643	−0.56 to 2.15	0.232
Diet						
Oral feeding	Reference			Reference		
L-tube feeding	8.18 ± 12.39	−17.93 to 34.29	0.518	6.63 ± 7.66	−9.52 to 22.79	0.398
Diabetes mellitus	−11.02 ± 14.18	−40.93 to 18.89	0.449	10.41 ± 8.78	−8.09 to 28.91	0.252
Heavy drinking	−1.80 ± 12.69	−28.57 to 24.98	0.889	2.03 ± 7.85	−14.53 to 18.59	0.799
HbA1c	−0.67 ± 4.85	−10.90 to 9.57	0.892	−2.88 ± 3.00	−9.21 to 3.45	0.350
CRP	0.55 ± 0.60	−0.71 to 1.81	0.371	−0.25 ± 0.37	−1.04 to 0.53	0.501

Values are standardized β coefficients (95% confidence interval) from multivariable linear regression analysis after adjustment for age, sex, NIHSS (National Institutes of Health Stroke Scale) score, and Charlson Comorbidity Index (CCI). Models were constructed separately for VL and GCM changes as dependent variables. No additional significant predictors—including lesion location (supratentorial vs. infratentorial) or stroke type (ischemic vs. hemorrhagic)—were identified. Abbreviations: CI = confidence interval.
